# Crosstalk between Vitamin D Metabolism, VDR Signalling, and Innate Immunity

**DOI:** 10.1155/2016/1375858

**Published:** 2016-06-15

**Authors:** Rui Lin

**Affiliations:** Department of Digestive Diseases, General Hospital, Tianjin Medical University, Tianjin 300052, China

## Abstract

The primary function of vitamin D is to regulate calcium homeostasis, which is essential for bone formation and resorption. Although diet is a source of vitamin D, most foods are naturally lacking vitamin D. Vitamin D is also manufactured in the skin through a photolysis process, leading to a process called the “sunshine vitamin.” The active form of vitamin D, 1,25-dihydroxyvitamin D (calcitriol), is biosynthesised in the kidney through the hydroxylation of 25-hydroxycholecalciferol by the CYP27B1 enzyme. It has been found that several immune cells express the vitamin D receptor (VDR) and CYP27B1; of the latter, synthesis is determined by several immune-specific signals. The realisation that vitamin D employs several molecular mechanisms to regulate innate immune responses is more recent. Furthermore, evidence collected from intervention studies indicates that vitamin D supplements may boost clinical responses to infections. This review considers the current knowledge of how immune signals regulate vitamin D metabolism and how innate immune system function is modulated by ligand-bound VDR.

## 1. Background

The actions of vitamin D are familiar in its classic capacity of mineral metabolism and bone health. Vitamin D promotes the intestinal absorption of phosphate and calcium, it stimulates the differentiation of progenitor cells to osteoclasts, and it recovers calcium from bones and encourages bone matrix mineralisation. Research into osteomalacia and rickets provided early evidence of the important role of vitamin D [1]. These diseases represent vitamin D deficiency and present symptoms of hypocalcaemia (low serum calcium levels) and skeletal deformity due to poor mineralisation of the bones [2]. Patients with these diseases usually have serum vitamin D levels below 20 nmol/L. To help reduce the incidence of rickets, infants in USA and in other countries typically receive daily vitamin D supplements of at least 200 IU (5 *μ*g). Although this strategy has reduced the incidence of rickets, it has not eliminated rickets, and rickets still persist [3]. Vitamin D deficiency is not limited to bone-related diseases but is also implicated in cardiovascular disease, autoimmune maladies such as type 1 diabetes mellitus, several cancers, inflammatory bowel disease, and multiple sclerosis [4].

The role of vitamin D, as it applies to human health, has undergone reevaluation following the discovery that VDR and CYP27B1 are expressed in cells such as the intestine, pancreas, prostate, and some immune cells, none of which are implicated in bone and mineral metabolism [4]. The biosynthesis of calcitriol by immune cells and peripheral tissue is of particular interest to immunology studies. This molecule is thought to modulate immune function in a manner similar to active cytokines [5]. This review outlines the role of vitamin D and its effects on the innate immune system.

## 2. Vitamin D Sources

There are three sources of vitamin D: UVB radiation-dependent endogenous production, dietary supplements, and nutritional sources. By far the most significant of vitamin D is UVB exposure and dietary supplementation is the least. There are several analogues of vitamin D; the two forms that are most relevant to human health are ergocalciferol (D_2_) and cholecalciferol (D_3_). The numbers of nonfortified foods that contain relevant quantities of either form of vitamin D are limited, fatty fish (mackerel, salmon, and sardines), cod liver oil, and some types of mushrooms, such as sundried shiitake [4, 6].

The conversion of 7-dehydrocholesterol to vitamin D_3_ occurs in the epidermal layer of skin. 7-Dehydrocholesterol maximally absorbs UVB radiation from the sun at wavelengths of ~300–325 nm, the presence of which is influenced by latitude, altitude, season, and cloud cover. For approximately six months of each year, at sea-level locations at latitudes of 45°, the UVB intensity is inadequate for vitamin D synthesis. This “vitamin D winter” extends at distances further away from the equator [7].

Dietary vitamin D intake is dependent upon a country's fortification policy and on an individual's dietary habits; by fortifying staple foods, such as dairy produce, some countries such as Canada and USA help to reduce vitamin D deficiency. In spite of these efforts, a global perspective review revealed that dietary supplements contribute 6–47% of vitamin D intake [8]. This suggests that where endogenous vitamin D production is low because UVB is insufficient, maintaining healthy levels of vitamin D is heavily dependent on supplements. As well as the factors identified earlier that determine UVB adequacy, an individual's endogenous vitamin D production is influenced by their genes, skin pigmentation, clothing, lifestyle, and use of sunscreen [9].

## 3. Vitamin D Biosynthesis and Sufficiency

**Figure 1:**
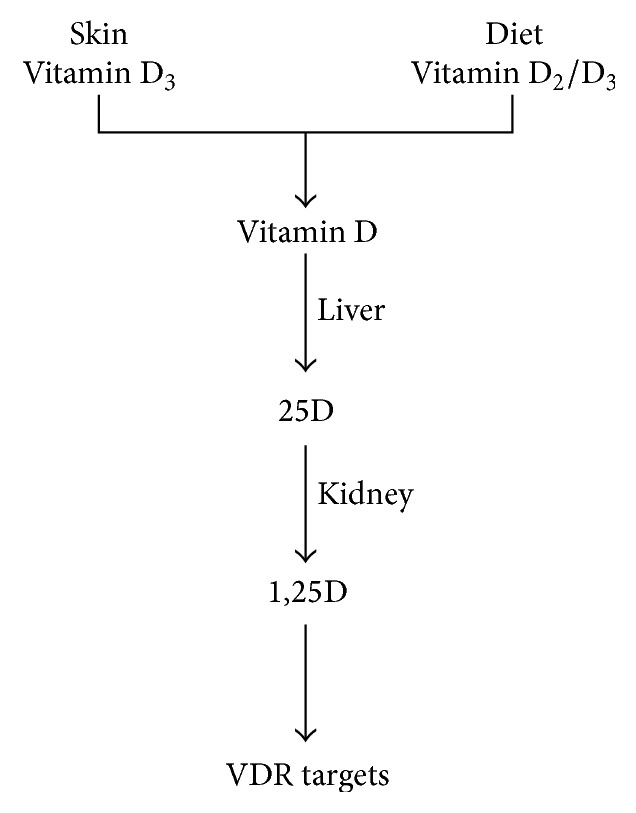
Vitamin D from skin and diet is converted to 25D in the liver and then converted to 1,25D in the kidney. 1,25D modulates the innate immunity system.

To become bioactive, cholecalciferol (D_3_) undergoes two modifications. In the first one, hepatic hydroxylating enzymes CYP2R1 and CYP27A1, and possibly others, catalyse D_3_ to produce prohormone 25-hydroxycholecalciferol (25D) [4]. This is the predominant circulating metabolite and it has a half-life of several weeks; it is used to determine vitamin D status. The prohormone undergoes 1*α*-hydroxylation by CYP27B1, resulting in its active form, 1,25-dihydroxyvitamin D (1,25D). This second modification occurs in the kidney, where regulated production is induced by parathyroid hormone (PTH) (Figure 1). It was originally thought that circulating levels of 1,25D were primarily derived from the kidney. Recent research has found that CYP27B1 expression is not limited to the kidney [10] and that other several tissues locally biosynthesise 1,25D, which acts intracellularly to regulate events within the cell or in a paracrine fashion. Through a negative feedback loop, 1,25D initiates the robust expression of* CYP24A1*, which codes for the CYP24 enzyme. This enzyme degrades 25D and 1,25D by hydroxylation of carbon 24, to generate biologically inactive metabolites.

Vitamin D deficiency is defined as circulating 25D levels below 20 ng/mL (50 nM) and insufficiency as 20–30 ng/mL (50–75 nM) [4]. Many researchers define sufficiency as levels of 30–32 ng/mL or higher. These levels were determined by the inverse relationship present between the circulating levels of 25D and PTH, the latter of which becomes deficient when 25D levels rise above 30 ng/mL. Yet a report from the Institute of Medicine defines sufficiency as ranging between 20 and 50 ng/mL (75–125 nM), with levels greater than 50 ng/mL (125 nM) considered to be excessive [11]. The report attracted controversy by concluding that few in the population suffered from vitamin D insufficiency or deficiency [12]. Furthermore, the report argued that there was a paucity of experimental evidence of vitamin D having important physiological functions beyond bone health. Without adequate randomised, placebo-controlled clinical trials investigating nonbone events, guidelines to establish recommended dietary intake could not be determined. Yet within recent years, there have been studies demonstrating that, in response to infection, immune system cells synthesise and respond to 1,25D. There is a growing body of clinical evidence collected from intervention trials showing that vitamin D sufficiency has prophylactic capability. Lastly, although vitamin D intoxication may occur, it is still not common and requires circulating levels to be in excess of 125 ng/mL [4]. Intoxication is typified by elevated blood levels of calcium.

## 4. The Vitamin D Receptors

The VDR is a member of the nuclear receptor superfamily. The VDR is activated by the binding of 1,25D [13]. Microarray expression profile studies have provided an enhanced understanding of vitamin D physiology and much can be explained by the VDR functioning as a gene transcription regulator [14]. The structure of the VDR incorporates an *α*-helical ligand-binding domain and a highly conserved DNA-binding domain [13]. 1,25D-liganded VDR forms a heterodimer with the retinoid-X receptor (RXR), which binds to vitamin D response elements (VDREs) in the regulatory area of the gene controlled by 1,25D.

VDREs are comprised of 5′-PuG(G/T)TCA-3′ (where Pu is any purine) repeat motifs separated by 3 bp (DR3) or everted repeats separated by 6 bp (ER6) or 8 bp (ER8) [13]. ER8 motifs recognised by VDR-RXR heterodimers encode the cytokine, interleukin-10 [15]. Transcription is initiated through the DNA-bound VDR/RXR complexes' recruitment of coregulatory proteins that instigate the necessary histone modification, chromatin remodelling, and RNA polymerase II binding [16]. Although several VDREs have been identified in locations close to the promoter regions [14], evidence from recent research indicates that DNA-bound VDR can operate over distances of 75 kbp to regulate target gene transcription [17]. Meanwhile, the VDR is able to suppress transcription, such as repressing cytokine gene expression in activated T-cells. In the presence of 1,25D, VDR/RXR heterodimers can dislodge DNA-bound nuclear factor (NF-AT), inhibiting cytokine expression. It has also been recently discovered that 1,25D-bound VDR interacts with FoxO transcription factors to suppress the cyclin D2 gene expression [18].

## 5. Vitamin D and Innate Immunity System

Reports of treating tuberculosis with cod liver oil presented the first evidence that vitamin D is a potential innate immune system stimulant [19]. Contemporary studies describe how calcitriol promotes the antimicrobial activity of macrophages and monocytes, which play a critical role in combatting pathogens such as* Mycobacterium tuberculosis*. The 1,25/VDR/RXR complex not only boosts the innate immune cells chemotactic and phagocytic capabilities, but also directly activates transcription of cathelicidin (hCAP18) and defensin *β*2 (DEFB) [20]. Following recognition of* M. tuberculosis*, through toll-like receptor signalling, monocytes induce CYP27B1 and VDR activity and directly modulate gene expression that favours cathelicidin production [21]. Other cytokines like interferon-*γ* or interleukin-4 also influence CYP27B1 expression [22]. Production of human cathelicidin (hCAP18), derived from LL-37, is upregulated in response to infection; it destroys microbial lipoprotein membranes [23].

Where infections are severe, there is an upsurge of neutrophils, which led to the original proposal of neutrophils being the primary source of cathelicidin [24]. This opinion has since been revised, as although neutrophils express VDR, they appear to lack the CYP27B1 capability required to convert 25D to 1,25D; this is essential to stimulate cathelicidin gene expression [22]. However, critically ill, septic patients have been found to have significantly lower serum 25D levels. This finding, derived from a cross-section analysis, showed a correlation between low serum 25D levels and reduced concentrations of cathelicidin [25]. This observation lends support to the hypothesised role of vitamin D regulating antimicrobial protein levels in a concentration-dependent manner and may be fundamental to infection control.

Vitamin D not only modulates monocytes, but also is important to other antigen presenting cells (APCs), especially dendritic cells (DCs). Calcitriol has also been credited with inhibiting the T cell cytokines, interleukin-2, and interleukin-17, as well as monocyte toll-like receptors [26]. A study of calcitriol supplements in healthy humans found that a high dose (1 *μ*g, twice a day for 7 days) led to the significant suppression of interleukin-6, a proinflammatory cytokine [27].

Dendritic cells express CYP27B1 which enables calcitriol and cholecalciferol to induce tolerogenic behaviour in these cells. The presence of the enzyme allows DCs to generate a high local concentration of calcitriol, which is needed to modulate immune responses. Data from* in vitro* studies using VDR and CYP27B1 knockout mice showed abnormal DC chemotaxis and a considerable increase in numbers of mature DCs [28]. In a placebo-controlled clinical trial of 95 tuberculosis patients, the inflammatory responses resolved quicker in patients that received a high dose of vitamin D and adjunctive therapy [29].

The gastrointestinal (GI) tract is a selectively permeable barrier that permits water and nutrient transport whilst inhibiting systemic pathogenic infection. Evidence from VDR knockout mice suggests that vitamin D has a role in regulating the GI tract barrier. The knockout mice showed a heightened vulnerability to lipopolysaccharides and chemically induced GI inflammation (DSS colitis) [30]. The integrity of the epithelial barrier was lost in the mice that had been exposed to DSS [30]. Compared to wild-type (WT) mice, VDR mice treated with DSS displayed a reduction in expression of E-cadherin, claudin-1, ZO-1, and occluding proteins [31]. In GI epithelial cells 1,25D stimulated transcription of E-cadherin [32]. The permeability of the gut increased in line with the loss of tight junction proteins in VDR knockout mice and vitamin D deficient mice [31]. Furthermore, elevated levels of inflammatory cytokines, such as TNF-*α*, were found to contribute to the loss of GI barrier integrity in vitamin D deficient and VDR knockout mice [30]. Together, the evidence from these studies indicates that vitamin D has an important regulatory role in maintaining the GI epithelium and its barrier function.

## 6. Molecular Mechanisms Underlying Vitamin D Regulate Innate Immunity

Investigating the immunomodulatory capability of vitamin D signalling is at the forefront of current research on developing the understanding of the mechanisms of vitamin D metabolism and 1,25D signalling as they apply to innate immune responses. It is known that CD14 expression is vigorously stimulated by 1,25D. CD14 is a TLR4 coreceptor that is required to recognise lipopolysaccharide.

With the completion of the Human Genome Project in 2003, the position of promoter-proximal consensus VDREs became known. The* in silico* screen pinpointed VDREs adjoining the transcription start-sites of genes encoding the antimicrobial peptides *β*-defensin 2 and cathelicidin [33]. Together with various cytokines and chemokines, antimicrobial peptides are amongst the first defensive mechanisms of the innate immune system to respond [34]. Immune responses may be enhanced by cathelicidin and some *β*-defensins that not only act against microbes, but also have chemoattractant capabilities, recruiting neutrophils, monocytes, and other immune cell molecules to the site of infection [34]. In the cell types investigated, expression of cathelicidin was robustly invigorated by 1,25D. A particularly interesting* in vivo* study looked at the regulation of cathelicidin peptide in the bile duct, which typically is microbe-free. The researchers noted that expression of the cathelicidin gene in the epithelial cells was regulated by the concentration of bile acids [35]. The probable contribution of VDR in the signalling is in accord with earlier studies, which indicate that VDRs have a bile acid sensing ability. Selecting animal models for research into cathelicidin and *β*-defensins needs to be made with care. The* CAMP *and* HBD2* are not conserved in mice, and, in humans and primates, the* CAMP* VDRE is embedded in an Alu repeat transposable element [20]. This particular VDRE-containing Alu repeat in the* CAMP* gene has only been found in the branch of primates that includes Old and New World monkeys, apes, and humans [36].

Cell-based investigations found that, in contrast to its effect on* CAMP* expression, the induction of* HBD2* expression by 1,25D alone was limited or absent [21]. Yet the robust expression of* HBD2* by interleukin-1*β* was doubled in the presence of 1,25D. It was later shown that signalling through TLR1/2 pattern receptors initiated interleukin-1*β* expression. For strong* HBD2* expression, both 1,25D and interleukin-1*β* were necessary [21]. It is probable that interleukin-1*β* signalling is mediated by NF-*κ*B transcription factor binding to the* HBD2* proximal promoter [37].

The importance of NF-*κ*B binding sites to innate immune signalling should not be underestimated. In a recent study, researchers discovered that ligand-bound VDR instigates expression of the genes for the nucleotide oligomerisation domain protein/caspase recruitment domain-containing protein (NOD2/CARD15), which is an encoding pattern recognition receptor [38]. NOD2 is an intracellular pattern recognition receptor, though structurally distinct from the archetypal TLRs. NOD2 is activated in response to muramyl dipeptide, which is a product of lysosome degradation of bacterial peptidoglycan.* NOD2* expression in epithelial and myeloid cells is vigorously induced by 1,25D through the VDR complex binding to distal high-affinity VDREs. NOD2 signalling also prompts NF-*κ*B activity and promotes* HBD2* expression [39]. Inducing NOD2 with 1,25D followed by muramyl dipeptide stimulated* HBD2* expression [38].

## 7. Conclusion and Future Research

Research over the past few years has confirmed that vitamin D has a role that extends beyond bone health and it is important for effective innate immune system responses. A wide range of tissue and cell types has been shown to express enzymes that metabolise vitamin D, which offers a reasonable mechanism for the autocrine, intracrine, and paracrine metabolism of cholecalciferol to the active calcitriol. As the understanding of the two-way interaction between vitamin D in its various forms and the immune system has grown, it has become apparent that vitamin D is fundamental in the innate immune system's response to microbial infections. Immune system dysregulation appears to be linked to vitamin D deficiency or adequacy. It is almost certain that there is more to discover about this interaction. However, it is important to bear in mind that species-specific differences can complicate research findings and many vitamin D-dependent mechanisms observed in humans may not be repeated in many animal models.

To date there is an absence of consensus as to what the recommended target serum level of vitamin D should be. It is also debated as to which vitamin D analogue is most beneficial as a dietary supplement, as different forms offer particular advantages to different immunomodulatory responses. To establish the effect of vitamin D supplements on the pathophysiology of various diseases requires a greater number of clinical trials involving more participants. Such trials may provide information on the effect of vitamin D upon the efficacy of other treatments as well as determining the optimal dosages and form. For now, the current evidence points to vitamin D being a relatively safe nutrient that offers promise in disease prevention and as an adjunctive therapy for immune-homeostasis impairment diseases.
